# Comment on Bilika et al. Applying Nociplastic Pain Criteria in Chronic Musculoskeletal Conditions: A Vignette Study. *J. Clin. Med.* 2025, *14*, 1179

**DOI:** 10.3390/jcm15103740

**Published:** 2026-05-13

**Authors:** Jelle C. Schouten, Frank J. P. M. Huygen, Jitske Tiemensma

**Affiliations:** Department of Pain Medicine, Erasmus Medical Center, 3015 GD Rotterdam, The Netherlands

We read with great interest the study by Bilika et al., which aimed to investigate the reliability and validity of the International Association for the Study of Pain (IASP) clinical criteria and grading system for nociplastic pain [1]. We would like to congratulate the authors on this important first step in evaluating the potential usefulness of the criteria for identifying nociplastic pain using clinical vignettes. We particularly appreciate the systematic and comprehensive development process of the vignettes. However, we would like to highlight two methodological aspects for which further clarification may strengthen the interpretation of the results.

First, the fourth step of the IASP clinical grading system, which corresponds to the seventh question in Supplementary Material S3 of Bilika et al., requires raters to assess whether there is clinically evoked pain hypersensitivity in the painful region [2]. To assess this criterion, raters therefore need explicit written information about the presence or absence of evoked pain hypersensitivity. However, Supplementary Material S1 of Bilika et al. includes only one vignette (example vignette no. 20) and does not provide this information. Consequently, it remains unclear how the raters assessed this criterion. This may affect the interpretation of the reported perfect (κ = 1.00) and almost perfect (κ = 0.93) inter-rater reliability in the first and second assessments, respectively. We note that some vignettes contain information about evoked pain hypersensitivity, as shown in Table 2 (Experts’ decisions) of Bilika et al., where revisions following expert disagreement refer to changes in sensitivity to palpation. However, if such information is not present in all vignettes, this criterion cannot be assessed consistently. Moreover, this is the only step in the grading system that explicitly requires physical examination. Even when written information is available, this step may be the least feasible to assess using clinical vignettes, which justifies the need for explicit discussion of this step in the manuscript.

Secondly, the authors state that they are investigating the criterion validity of the IASP criteria. To do so, they use the clinical criteria and grading system of the IASP as the index test and the vignette classification by a panel of experts as the reference standard. Criterion validity is defined as “the correlation of a scale with some other measure of the trait or disorder under study, ideally, a ‘gold standard’ that has been used and accepted in the field” [3]. Given the absence of a gold standard and a generally accepted reference standard, the use of a panel of experts is both understandable and common in diagnostic accuracy studies [4]. However, we believe that further clarification regarding the independence of the reference standard from the index test would be valuable. The panel of experts consisted of three physiotherapists who based their assessments on scientific evidence and clinical expertise, and provided transparent evaluations that were subject to critical appraisal and discussion. Despite these safeguards, both the index test and the reference standard ultimately reflect expert-opinion judgements that were likely based on the same underlying vignette information and evidence. The IASP criteria themselves are evidence-informed and largely correspond to the variables that the expert panel likely used in its deliberations. These variables include widespread pain, the absence of nociceptive and/or neuropathic mechanisms fully responsible for the pain, and clinical signs of pain hypersensitivity in the affected area, as described in the literature on discriminating nociplastic pain from other pain mechanisms [5,6]. If both the index test and the reference standard rely on the same evidence-based variables to determine whether pain is nociplastic, incorporation bias may arise, potentially inflating the apparent diagnostic accuracy of the IASP criteria [7,8]. Although a panel of experts may currently be the most feasible comparator for assessing criterion validity, we question whether criterion validity can be meaningfully established in the absence of an independent reference standard. We therefore believe that these findings may be more appropriately interpreted as agreements between the raters and an expert-derived vignette classification than as conclusive evidence of criterion validity.

We would appreciate further clarification from the authors about how the criterion for evoked pain hypersensitivity was assessed, as this information did not appear to be explicitly stated in all vignettes. Additionally, we would appreciate further explanation about how the authors ensured independence of the index test and the reference standard, given the likely overlap in evidence-based discriminating variables. Clarification of these issues would strengthen the interpretation of the results and their implications for the IASP clinical criteria and grading system for nociplastic pain.
